# A holistic and sustainable approach linked to drought tolerance of Mediterranean crops

**DOI:** 10.3389/fpls.2023.1167376

**Published:** 2023-06-15

**Authors:** Maurizio Trovato, Faiçal Brini, Khalil Mseddi, Sophia Rhizopoulou, Matthew Alan Jones

**Affiliations:** ^1^ Department of Biology and Biotechnologies, Sapienza University of Rome, Rome, Italy; ^2^ Biotechnology and Plant Improvement Laboratory, Centre of Biotechnology of Sfax (CBS), Sfax, Tunisia; ^3^ Faculty of Sciences of Sfax, University of Sfax, Sfax, Tunisia; ^4^ Department of Biology, National and Kapodistrian University of Athens, Athens, Greece; ^5^ School of Molecular Biosciences, University of Glasgow, Glasgow, United Kingdom

**Keywords:** global warming, drought tolerance, crop yield, ABA, genome editing, wild plants

## Abstract

The rapid increase in average temperatures and the progressive reduction in rainfalls caused by climate change is reducing crop yields worldwide, particularly in regions with hot and semi-arid climates such as the Mediterranean area. In natural conditions, plants respond to environmental drought stress with diverse morphological, physiological, and biochemical adaptations in an attempt to escape, avoid, or tolerate drought stress. Among these adaptations to stress, the accumulation of abscisic acid (ABA) is of pivotal importance. Many biotechnological approaches to improve stress tolerance by increasing the exogenous or endogenous content of ABA have proved to be effective. In most cases the resultant drought tolerance is associated with low productivity incompatible with the requirements of modern agriculture. The on-going climate crisis has provoked the search for strategies to increase crop yield under warmer conditions. Several biotechnological strategies, such as the genetic improvement of crops or the generation of transgenic plants for genes involved in drought tolerance, have been attempted with unsatisfactory results suggesting the need for new approaches. Among these, the genetic modification of transcription factors or regulators of signaling cascades provide a promising alternative. To reconcile drought tolerance with crop yield, we propose mutagenesis of genes controlling key signaling components downstream of ABA accumulation in local landraces to modulate responses. We also discuss the advantages of tackling this challenge with a holistic approach involving different knowledge and perspectives, and the problem of distributing the selected lines at subsidized prices to guarantee their use by small family farms.

## Introduction

The combined effects of climatic change, overpopulation and unregulated urbanization are reducing water availability in large areas of the world, leading to severe crop losses. The impact of global warming varies across geographic regions, with issues especially prevalent in regions with hot and semi-arid climates such as some Mediterranean countries, where the average temperatures are expected to increase by 20% by the end of the 21st century, with dramatic effects on crop yields and the socio-economic conditions of these regions (Hilmi et al., 2022). The Middle East and North Africa (MENA) region is ranked as the driest region in the world (The World Bank, 2018), with only 1.4% of the world’s available fresh water (Roudi-Fahimi et al., 2000). In this context, climate change in Mediterranean MENA regions is expected to proceed faster and with more negative economic effects due to the combination of low rainfall, overpopulation, and agricultural economies based on small family farms (Borghesi and Ticci, 2019). Tunisia, for example, experienced five major drought events in the last decade, reducing yields by up to 50% (World Food Programme, 2011) and leading to spikes in wheat imports. Furthermore, according to the World Bank (The World Bank, 2021) the average temperature is expected to increase by 2°C by 2030, with an estimated loss of cereal production of 30%.

The negative economic effects caused by climate change are particularly severe in countries where agriculture significantly contributes to Gross Domestic Product (GDP) and employment. Since primary food production is the most critical node of the food supply chain, the reduction in crop yield caused by climate change can cause dramatic effects on subsequent nodes of the chain leading to food shortages, higher food prices, loss of employment and political instability. As noted by the FAO report “The State of Food and Agriculture 2021” (FAO, 2021) small-family farms of low-income regions are particularly vulnerable to stresses reducing primary crop production and personal income. This situation can lead to increased poverty and migration, further weakening the economic structure and contributing to poltical instability. Furthermore, climate change-induced crop yield reduction can indirectly affect human health, leading to food insecurity, malnutrition and, in turn, acute and chronic diseases with high social and economic costs.

The traditional strategy to cope with this problem relies upon crop breeding, which has led to some promising improvement and the identification of numerous SNPs (Single Nucleotide Polymorphisms), and QTLs (Quantitative Trait Loci) associated with drought resistance or productivity (Hussain et al., 2017; Tahmasebi et al., 2017). Unfortunately breeding programs are slow, expensive, and laborious and are falling behind the fast pace of global warming. Due to a widespread belief of the scientific community that engineering single genes involved in stress response would have generated drought-tolerant crops, several transgenic plants overexpressing these genes have been created as a popular alternative to breeding programs (Brini et al., 2007a; Brini et al., 2007b; Carraro and Di Iorio, 2022). Although several studies have reported the successful generation of transgenic crops more tolerant to environmental stresses, in most of the cases they did not provide information on productivity, and in some cases even reported a reduction in some yield indicators, such as plant height and number of seeds, especially under unstressed conditions (Mansour and Ali, 2017; Forlani et al., 2019). Proline accumulation, for example, is commonly associated with stress tolerance (Trovato et al., 2008) and several papers report increased drought or salt tolerance in plants overexpressing proline biosynthesis genes (Per et al., 2017). However, commercially-available crops overexpressing proline synthesis genes are not known to date, and a work specifically designed to evaluate the effect of proline on yield did not find convincing evidence that proline can improve seed production under stress (Mattioli et al., 2020).

Considering the complexity of the environmental stresses a plant can be exposed to in Africa, any attempt to improve plant tolerance by modifying single genes may be overly simplistic. Drought stress, in particular, is particularly complex and variable, in terms of intensity, duration and timing of action, and is known to induce the expression of many genes and signaling pathways (Lawlor, 2012). Accordingly, a multi-gene transformation strategy achieved by genetic modification of transcription factors or regulators of signaling cascades seems more promising than targeting single genes for improving drought resistance (Fang and Xiong, 2015). In line with these considerations, HB4®, the only drought-tolerant wheat approved for growth, consumption and commercialization in some countries relies on the stress-inducible expression of the homeo-domain-leucine zipper (HD-zip) transcription factor (TF) HaHB4 from sunflower (*Helianthus annuus*), which can bind to promoters containing the cognate recognition element to regulate the expression of numerous downstream genes (Bergau, 2019). Overall, despite the urge to develop better-adapted crops to a changing climate, the relatively modest results achieved so far leave room to develop novel and more sustainable strategies to produce productive crops under drought conditions and affordable for smallholder farmers in Africa. To be effective, these strategies should identify drought-resistant genes in local landraces, carry out random mutagenesis with TILLING or CRISPR/Cas9, and co-select lines with drought tolerance and good productivity. Finally, actions should be taken to guarantee small farmers free or subsidized distribution of the selected lines.

## Defining how plants cope with environmental stresses

It is worthwhile to look at the multiple strategies developed by plant species to survive drought stress. Drought is a complex trait encoded by many genes, the majority of which contribute only a minor genetic contribution. The effects of drought on plants may be different depending on drought duration and intensity, on the developmental phase that stress is perceived by the plants, and on the type of drought stress. Although drought is generally defined as a prolonged period of water deficit, usually because of rainfall absence, its effects overlap those caused by water shortage, occurring when transpiration exceeds water uptake, but also under conditions of salinity or osmotic stress (Bray, 1997). Because of this coincidence, it is common practice to simulate drought in laboratory conditions using high PEG (Polyethylene glycol) concentrations in liquid or solid media. The results achieved in the laboratory, however, are seldom confirmed in field conditions which makes it difficult to apply these advances in the field.

**Figure 1:**
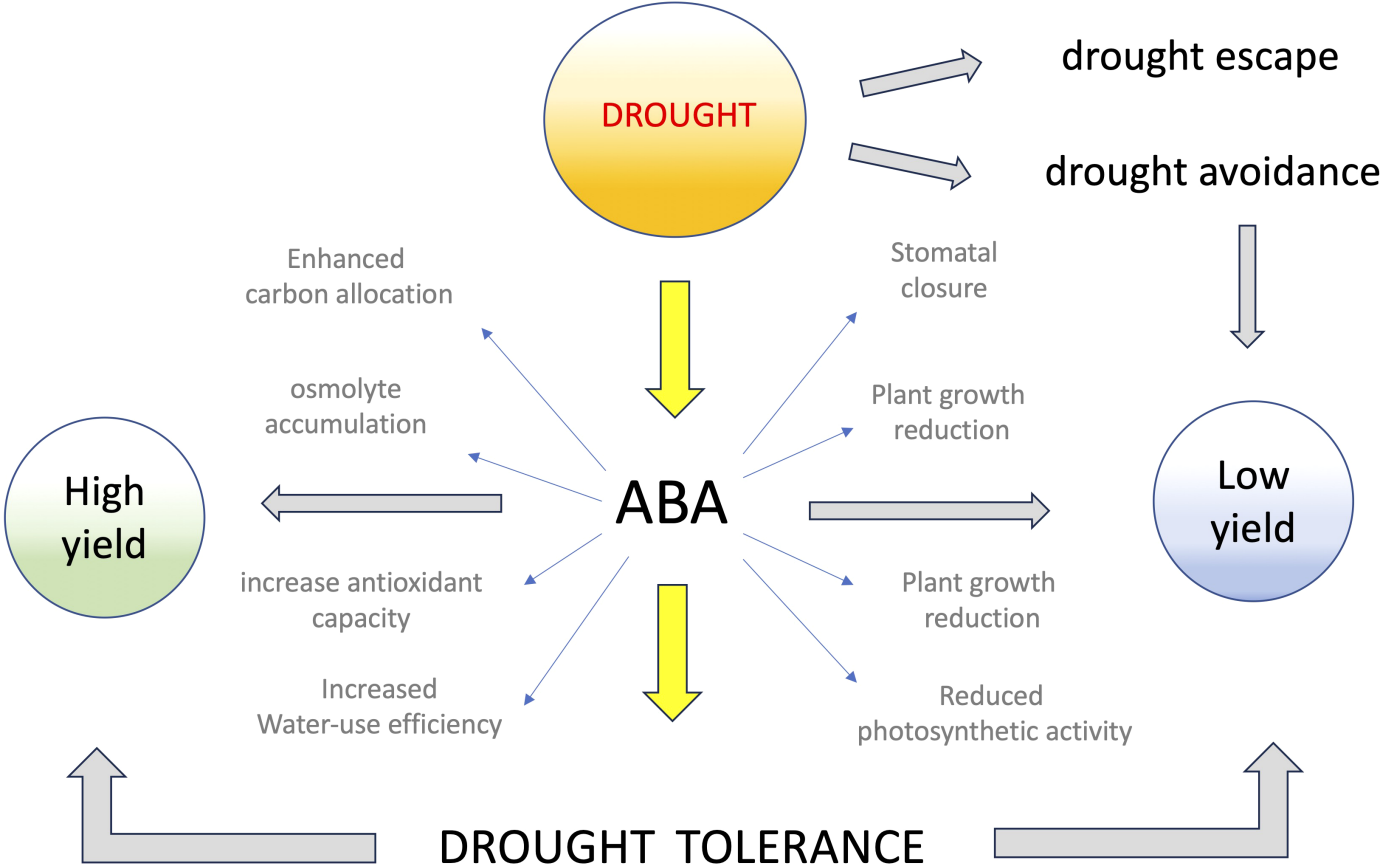
Main mechanisms of drought resistance and effects of ABA on crop yield. Plants try to escape, avoid or tolerate drought stress through various morphological, physiological, and biochemical adaptations, including accumulation of ABA, which triggers different signaling pathways leading to drought tolerance and, often, low productivity. ABA-induced drought tolerance, however, can be associated with high productivity, possibly due to improved water use efficiency, carbon allocation, and osmolytes accumulation.

In natural conditions, plants respond to environmental drought stress with diverse morphological, physiological, and biochemical adaptations (Bohnert et al., 1995), trying to escape, avoid, or tolerate drought stress (**Figure 1**). Drought escape is typically obtained through early flowering (Rhizopoulou and Pantazi, 2015), which allows the plant to quickly set a few seeds and survive by accelerating the life cycle (Dolferus, 2014). A good example of drought escape is found in resurrection plants, such as *Craterostigma plantagineum* or *Selaginella lepidophylla*, which can survive for months without water, but flower and set seeds in a few days when occasional rains fall (Scott, 2000). Drought avoidance tries to reduce water loss by preventing excessive transpiration with morpho-physiological adaptations such as covering the leaves with wax cuticles or transforming leaves into thorns. Some halophyte plants, such as the wild crop *Trifolium fragigera*, provide examples of drought avoidance that can attain by removing salt excess by secretion or compartmentalization into vacuoles (Jēkabsone et al., 2022). In addition, some wild plant species, e.g. caper (*Capparis spinosa*) and carob (*Ceratonia siliqua*), possess ecophysiological strategies to cope with water shortage and exhibit growth and productivity during and after the dry summer period in the Mediterranean region (Rhizopoulou, 1990; Rhizopoulou and Davies, 1991; Rhizopoulou, 2004; Rhizopoulou and Kapolas, 2015). Drought tolerance, on the contrary, attempts to permit plant life even in the absence of sufficient water supply, usually by accumulating compatible osmolytes or enhancing the antioxidant plant’s capacity (Bartels and Sunkar, 2005), or by delaying flowering and slowing down metabolism until favorable conditions return (Syed et al., 2015). Some members of the halophyte family, such as *Thellungiella* or *Salicornia*, capable of accumulating high cellular levels of compatible osmolytes, are good examples of drought-tolerant plants (Mishra and Tanna, 2017). Most of the mechanisms evolved to improve drought tolerance in wild plants are associated with strong reductions in seed yield, which is limited to offspring survival and is much lower than under favorable environmental conditions (**Figure 1**). Thus, while it is important to study the mechanisms underlying drought tolerance of these plants to identify the relevant genes involved, we must be cautious in mimicking their strategies not to risk limiting productivity.

## ABA is involved in drought tolerance, which is often associated with low productivity

Many phytohormones, such as abscisic acid (ABA), auxin, cytokinins, gibberellins, ethylene, and jasmonic acid, as well many compatible osmolytes like proline, γ-aminobutyric acid (GABA), and sugars accumulate after stress and are thought to contribute to tolerance, but ABA, universally considered as the stress hormone, seems to play a pivotal role in stress tolerance. Irrespective of the type of drought a plant experiences, the ultimate consequence of the stress is cell dehydration, and, soon after, ABA accumulation, which, in turn, triggers diverse signaling pathways to activate downstream adaptive responses, such as stomatal closure, improved hydraulic conductivity, improved photosynthetic activity, organized soil microbial communities, activation of transcriptional and post-transcriptional gene expression, metabolic alterations, root elongation, and stimulation of antioxidant capacity (Todaka et al., 2019). The strict correlation between ABA accumulation and water deficiency strongly suggests that this phytohormone can play a key role in establishing drought tolerance in plants (Yoshida et al., 2014; Puértolas et al., 2017). Indeed, several studies have reported increased drought tolerance in plants treated with exogenous ABA (Ali et al., 2020) or synthetic ABA analogs (Zhou et al., 2019), as well as in transgenic plants expressing genes involved in ABA signaling (Fujii and Zhu, 2009).

Despite this promise, ABA-induced drought tolerance is often associated with low levels of crop productivity. Since negative or ambiguous results are usually not published, this concept is underestimated but only indirectly supported by the lack of transgenic lines or working protocols currently available in the market or used by farmers. A few examples of drought-tolerant plants with no yield gain, though, can be found here and there in the scientific literature, as in He et al. (2019), who reported improved drought tolerance but no yield benefit in soybean plants treated with exogenous ABA. Given our fundamental understanding of ABA signaling, it is likely that ABA-induced stomatal closure limits water loss by evapotranspiration, but also carbon dioxide entry, reducing carbon assimilation and photosynthesis efficiency (Lamaoui et al., 2018). The problem of reconciling drought tolerance with crop yield, especially in field conditions, holds true for transgenic plants transformed with other stress-related genes or treated with other exogenous substances, such as proline, sugars, and plant hormones, and remains the biggest challenge to tackle.

## Alternative approaches to reconcile drought tolerance with crop yield

Considering the number of physiological traits contributing to yield under drought stress and potentially modulated by ABA signaling (e.g. relative water content, water-use efficiency, transpiration efficiency, crop growth and partitioning rate, root traits, osmolyte accumulation), the targeted mutagenesis of ABA signaling modulators followed by co-selection of high-yielding and drought-tolerant lines seems a promising strategy. Instead of focusing on drought tolerance in the vegetative phase (by measuring indicators not necessarily correlated with grain yields, such as leaf water potential or osmotic adjustment), variability in genes involved in drought tolerance should be generated and analyzed at the reproductive phase to select the lines most productive under drought conditions. To optimize crop productivity, high-performance lines should be selected in both drought and well-hydrated conditions to avoid the frequently reported problem of plants performing well under stress but poorly without stress. A more comprehensive approach to mutant selection will be vital, including molecular biology, botany, ecophysiology, and crop breeding expertise.

## Genome editing of ABA signaling in durum wheat

Under optimal growing conditions, the levels of productivity of modern crops are not comparable with that of their wild relatives, although a few species perform better in drought conditions and could serve as a reservoir of genetic and phenotypic diversity (Kiani et al., 2015; Rafique et al., 2017; Mammadov et al., 2018). A good example is the wild emmer wheat (*Triticum dicoccoides*), a naturally drought-tolerant species that can set fertile seeds even in desert environments (Khan et al., 2022). By comparing the most high-yielding, drought-tolerant wild species, with phenomic and genome-wide techniques, we could discover common traits and genes associated with productivity to be used for genetic improvement. As a proof-of-concept, we chose to generate mutations leading to constitutive ABA activation and drought tolerance, to select lines with the best possible yield under suitable drought conditions.

**Figure 2:**
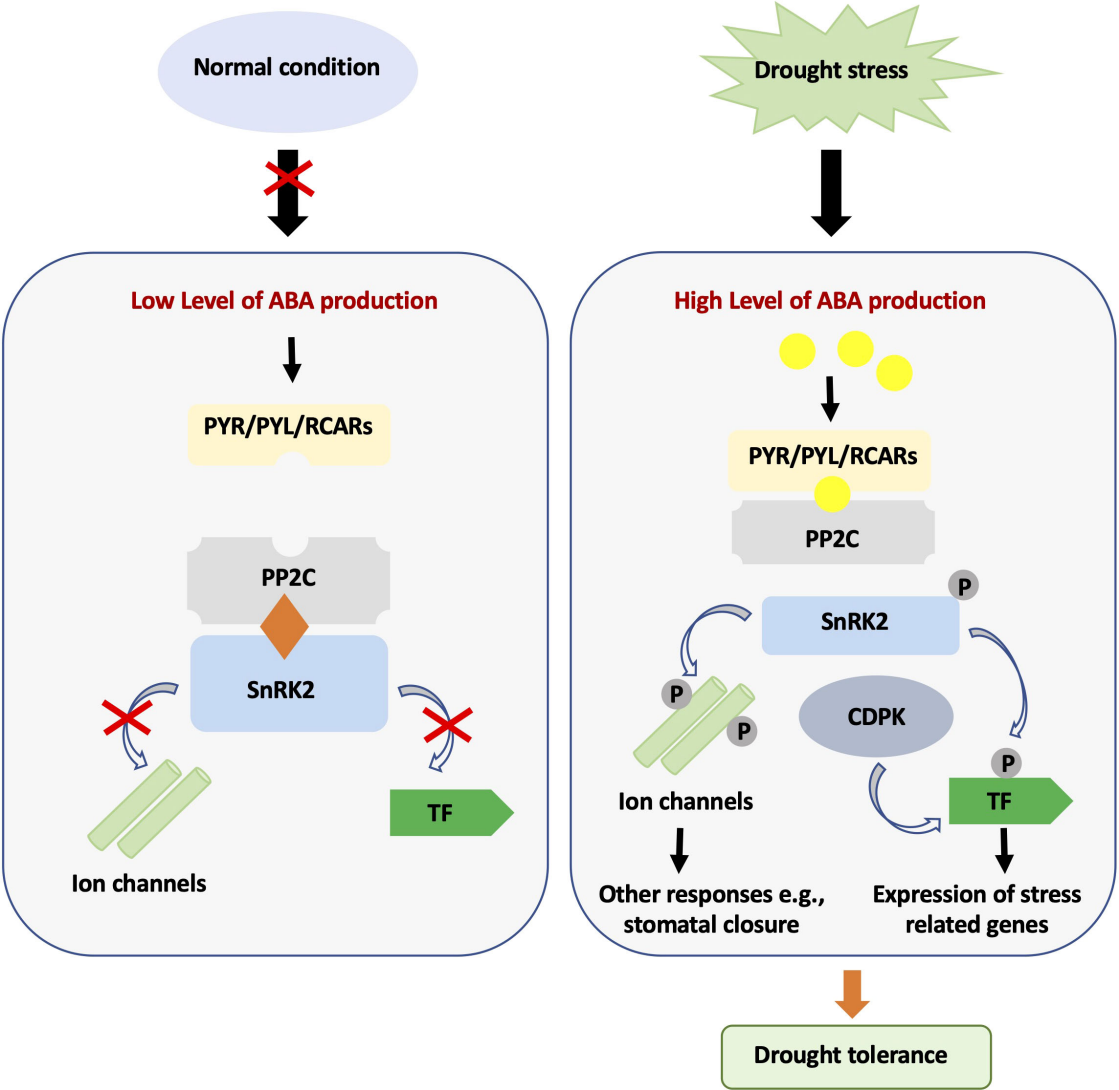
ABA-mediated signaling pathway during normal and drought stress conditions. Under normal conditions, ABA content is low, and SnRK2 protein kinase activity is inhibited by PP2C phosphatases. Under drought stress, the cellular ABA level increases, and ABA then binds to PYR/PYL/RCARs, which in turn bind and inactivate PP2Cs. The SnRK2s autoactivate when they dissociate from PP2Cs. Activated SnRK2s phosphorylate downstream targets and trigger ABA-induced physiological and molecular responses.

We think that genes involved in drought-related signaling pathways are better suited to cope with the complexity of dehydration stress. Indeed, drought and water stress have profound effects on plant life and elicit correspondingly complex physiological responses, many of which are induced by ABA accumulation. In a transcriptome analysis of *Arabidopsis* plants subjected to moderate dehydration stress, for example, Urano et al. (2017) found upregulation of 5271 genes and downregulation of 6303 genes. Most of these genes were found to code for proteins indirectly involved in drought tolerance, such as genes for osmolyte biosynthesis and catabolism, antioxidant genes, sugars, chaperones, heat shock proteins, and late embryogenesis abundant (LEA) proteins. A second class of genes was found to encode proteins involved in stress signaling, such as protein kinases, protein phosphatases, transcription factors, and proteins involved in protein degradation (Urano et al., 2017). In response to drought, ABA levels quickly increase in vegetative tissues leading to the activation of PYRABACTIN RESISTANCE1 (PYL)/PYR1-LIKE (PYR) ABA receptors and inhibition of group A PP2C phosphatase. In turn, PP2C inhibition allows autophosphorylation and activation of the SUCROSE NONFERMENTING 1-RELATED SUBFAMILY2 (SnRK2) kinase and subsequent phosphorylation of multiple downstream targets (**Figure 2**; Yoshida et al., 2014). As expected, ABA-mediated responses to drought are complex and involve hundreds of genes. Microarray analysis of *sal1* mutant, a phosphatase involved in retrograde signaling and constitutive activation of ABA signaling in *Arabidopsis* (Wilson et al., 2009), for example, revealed altered expression of more than 1800 genes, including heat shock proteins, transcription factors, aquaporins, light-inducible proteins, stress signaling kinases, stress-inducible proteins and antioxidant enzymes (Wilson et al., 2009). In addition, SAL1 has been shown to alter the circadian system (Litthauer et al., 2018), affecting the recently discovered relationships between stress response and the circadian clock and potentially allowing a better modulation of the plant’s response to the actual drought levels.

To select drought-tolerant, well-productive wheat varieties, ABA signaling mutants should be first identified for drought tolerance and then selected for productivity under drought conditions. The use of CRISPR/Cas mutagenesis is key to accelerating genetic improvement, especially for cereal crops, which have large and polyploid genomes, and long generation times. Importantly, the use of imprecise non-homologous end-joining (NHEJ) recombination by CRISPR/Cas9, and the possibility to introduce multiple gRNAs in the plant genome is expected to widen the genetic variability with beneficial effects in the process of selection (Jaganathan et al., 2018). Last but not least, CRISPR/Cas9 is gaining increasing popularity in crop research and is socially more accepted than transgenic plants, although full acceptance by public regulatory organizations is still awaited.

## Development of sustainable solutions for African farmers

Maintaining high crop yields in drought conditions can be achieved or supported in many ways, such as using sophisticated irrigation methods guided by artificial intelligence (AI), drones, and satellite systems, desalting seawater, or using automated shelters and fertilizations. However, all these methods require technological skills and high economic and energy costs that are difficult for small rural farmers to bear. The introduction of new crop varieties capable of good yields in arid climates, combined with good practices of conservation agriculture, on the contrary, may be a practical solution suitable for small family farms, provided the lines are distributed for free or at low cost. The generation of high-yielding drought-resistant plants adapted to increasingly hotter and drier climates is a long and expensive process in itself that needs to be reconciled with economic and environmental sustainability and adapted to specific local conditions. This objective is particularly significant for countries where agriculture is based on many small family farms. Provided the proof-of-concept is successful, it will be pivotal to search for local wild crops or landraces to edit the genomes of varieties most suited to local conditions. Accordingly, a close collaboration between molecular biologists, botanists, ecophysiologists, and policy-makers is required for the effective development of highly productive crops resistant to specific local conditions and freely available to local stakeholders.

Another potential problem is the number of registered patents on CRISPR/Cas9 technology which could translate into royalties or commissions at unsustainable costs for small farmers. From a scientific point of view, the identification of genetic determinants capable of good yields under drought conditions is a complex task that justifies the use of multiple strategies, including CRISPR/Cas9. Once the genes and signaling pathways involved in drought tolerance are known, it will be possible to improve crop yield with less problematic tools, such as precision breeding or TILLING. From a socio-economic point of view, on the other hand, it is desirable that governments and policymakers can tackle the long-standing problem of the intellectual and commercial protection of patents. Faced with possible humanitarian crises, such as world hunger, global warming, and the COVID pandemic, has already happened that humanitarian organizations or sovereign governments have stepped in to cover the costs associated with patent-holding companies to address these issues. Given the context, such an eventuality could well occur for many countries in North Africa and the sub-Saharan belt to prevent the spread of socio-political instability. Research and educational use of these technologies are already possible, and some academic subjects are pushing for complete liberalization. Wageningen University and Research in the Netherlands, one of the major patent holders of CRISPR technology, has recently announced that it will allow non-profit organizations to freely use its CRISPR/Cas9 gene-editing technology for non-commercial applications in food and agriculture (Nature Editorial, 2021). Overall, this perspective highlights the potentiality of targeting ABA signaling genes through CRISPR/Cas9 mutagenesis, which seems a promising strategy to select high-yielding, drought-tolerant crops, provided collaborative efforts and equitable access to genetic resources is guaranteed.

## Data availability statement

The original contributions presented in the study are included in the article/supplementary material. Further inquiries can be directed to the corresponding author.

## Author contributions

All authors listed have made a substantial, direct, and intellectual contribution to the work and approved it for publication.
